# Long non-coding RNA VIM Antisense RNA 1 (VIM-AS1) sponges microRNA-29 to participate in diabetic retinopathy

**DOI:** 10.1007/s00592-020-01536-2

**Published:** 2020-05-23

**Authors:** Feng Zeng, Gang Luo, Yamei Lu, Zhaotian Zhang, Yuanqing Zhou, Yonging Chen, Zhiyan Zhou

**Affiliations:** 1 Department of Ophthalmology, Qingyuan People’s Hospital, The Sixth Affiliated Hospital of Guangzhou Medical University, Qingyuan City, 511500 Guangdong Province People’s Republic of China; 2 Department of Critical Care Medicine, Qingyuan People’s Hospital, The Sixth Affiliated Hospital of Guangzhou Medical University, Qingyuan City, 511500 Guangdong Province People’s Republic of China; 3 Zhong Shan Ophthalmological Center, Sun Yat-Sen University, Guangdong, 510000 People’s Republic of China

**Keywords:** Diabetic retinopathy, VIM-AS1, T2D, Retinal pigment epithelial cell, Apoptosis, miR-29

## Abstract

**Aims:**

Long non-coding RNA (lncRNA) VIM Antisense RNA 1 (VIM-AS1) has been reported to be correlated with type 2 diabetes (T2D) susceptibility, while the roles of this lncRNA in T2D and its complications remain unclear. This study aimed to explore the role of VIM-AS1 in diabetic retinopathy (DR).

**Methods:**

Gene expression levels in both human specimens and in vitro cultivated cells were determined by qPCR and western blot. Overexpression experiments were performed to analyze gene interactions. Cell apoptosis after transfections was detected by cell apoptosis assay.

**Results:**

We found that VIM-AS1 was significantly downregulated in T2D patients in comparison with that in healthy controls. Specifically, the expression levels of VIM-AS1 were lowest among T2D patients complicated with DR. Bioinformatics analysis showed that VIM-AS1 can interact with microRNA 29 (miR-29), which is a critical player in high glucose-induced apoptosis of human retinal pigment epithelial cells (RPEs). Dual-luciferase assay also revealed the direct interaction between them. High glucose treatment led to upregulated miR-29 and downregulated VIM-AS1. However, overexpression of VIM-AS1 and miR-29 did not affect the expression of each other. Cell apoptosis analysis showed that overexpression of VIM-AS1 reduced the enhancing effects of miR-29 overexpression on RPEs cell proliferation.

**Conclusions:**

Therefore, VIM-AS1 may sponge miR-29 to participate in DR.

## Introduction

Diabetes is the most commonly diagnosed metabolic disorder in clinical practice [1]. With the changes in life style as well as the growing of aging population, incidence of diabetes among adults (> 18 years) has increased from 4.7 to 8.5% in the past 3 decades [1]. It is predicted that incidence of this disease will be continuously increasing until 2050 [2, 3]. Diabetes itself in most cases is not lethal, while diabetic patients have a 50% higher risk of deaths from any other severe clinical disorders in comparison with people without diabetes [2]. One reason of the high mortality rate is that the high glucose environment in the body of patients can induce damages to almost all important organs, including kidney, heart, stomach and eyes [4]. Diabetic eye, or diabetic retinopathy (DR), affects about one-third of type 2 diabetes (T2D) patients and is a major cause of vision loss in adults [5]. Even with active treatment, blindness will inevitably occur in 10% of the patients [6]. Therefore, novel therapies are still of great importance.

The development of diabetic complications including DR involves multiple signaling pathways [7], and the characterization of these molecular pathways is critical for the development of novel targeted therapies [8]. It has been well established that non-coding RNAs (ncRNAs), such as miRNA and long (> 200 nt) ncRNAs (lncRNAs), are critical players in the pathogenesis of DR [9]. Some miRNAs, such as miR-29, can regulate eye cell apoptosis under high glucose environment to participate in the disease progression [10]. VIM-AS1 is a recently identified cancer-related lncRNA in colon cancer [11]. A recent study reported that the expression of VIM-AS1 was also altered in T2D [12]. Our bioinformatics analysis showed that VIM-AS1 may interact with miR-29. This study was therefore carried out to investigate the interaction between VIM-AS1 and miR-29 in DR.

## Materials and methods

### Research subjects

A total of 60 patients with DR (40 males and 20 females, age range 38–66 years old, mean age 49.1 ± 5.8 years old), 60 patients with diabetic nephropathy (DN, 40 males and 20 females, age range 39–67 years old, mean age 49.3 ± 5.9 years old), 60 patients with diabetic gastroparesis (DG, 40 males and 20 females, age range 39–67 years old, mean age 49.8 ± 6.0 years old), diabetic ulcers (DU, 40 males and 20 females, age range 38–66 years old, mean age 49.5 ± 6.1 years old), T2D patients with complications (T2D, 40 males and 20 females, age range 38–66 years old, mean age 49.7 ± 6.1 years old) and 60 healthy controls (control, 40 males and 20 females, age range 38–66 years old, mean age 49.7 ± 6.1 years old). All participants were enrolled at the Sixth Affiliated Hospital of Guangzhou Medical University between March 2016 and March 2019. All patients were informed of the experimental principle, and they all signed the informed consent. This study was approved by the Ethics Committee of aforementioned hospital before the admission of participants.

### Plasma and cells

Under fasting conditions, blood (5 ml) was extracted from each participant before the initiation of therapies. Blood samples were injected into EDTA-treated tubes, followed by centrifugation for 10 min at 1200 g to prepare plasma samples. The human retinal pigment epithelial cell (RPE) cell line H1RPE7 was purchased from Sigma-Aldrich (USA). Cells were cultivated under conditions of 95% humidity, 5% CO_2_ and 37 °C. Cell culture medium was composed of 90% DMEM: F12 medium and 10% FBS.

### Transient transfections

VIM-AS1 expression vector was constructed using pcDNA3.1 vector as backbone. NC (negative control) miRNA and miR-29 mimic were synthesized by GenePharma (Shanghai, China). DNAs were mixed with Invitrogen Lipofectamine 2000 Transfection Reagent (Thermo Fisher Scientific) to a ratio of 1: 3 (μg to μl), followed by the incubation with 10^6^ cells at 37 °C for 6 h. After that, cells were washed with fresh medium to terminate transfections and avoid cytotoxicity. Transfection of empty vector or NC miRNA was NC group. Untransfected cells were used as control (C) cells. The following experiments were performed using cells harvested at 24 h post-transfection.

### Dual-luciferase reporter assay

Through aforementioned methods, different transfection combinations: (1) VIM-AS1 expression vector + NC miRNA (NC group); (2) VIM-AS1 expression vector + miR-29 mimic (miR-29 group) were transfected into h1RPE7 cells. It is worth noting that psiCHECK-1 vector (Promega) was used as the backbone to construct VIM-AS1 expression vector in this assay. Relative luciferase activity was normalized using firefly luminescence.

### RNA extractions and DNA digestion

Total RNA extractions were performed using TRIzol Plus RNA purification kit (Thermo Fisher Scientific). To harvest miRNAs, 85% ethanol was used for RNA precipitation and washing. The gDNA Eraser (TaKaRa, Japan) was used to digest all RNA samples to remove genomic DNAs. In cases of D-glucose treatment, h1RPE7 cells were cultivated in medium containing 5, 10, 20 and 30 mM D-glucose for 24 h before RNA extraction.

### qRT-PCR

Reverse transcriptions (RTs) were performed using PrimeScript RT reagent kit (TaKaRa, Japan) with RNA samples as templates and poly (T) as primer. The synthesized cDNA samples were used as template to prepare qPCR mixtures using QuantiNova SYBR Green PCR Kit (QIAGEN). Expression levels of VIM-AS1 were normalized to GAPDH endogenous control. To measure the expression levels of mature miR-29, all-in-oneTM miRNA qRT-PCR Detection Kit (GeneCopoeia, Guangzhou, China) was used to perform all steps, including addition of poly (A), RTs and qPCR reactions. Default threshold settings were used to determine threshold cycle (CT) values, and data normalization was performed using 2^−ΔΔCT^ method.

### Cell apoptosis assay

Cell apoptosis assay was used to monitor the effects of transfection on cell apoptosis. In brief, 2-ml cell suspension containing 10^5^ cells was added into each well of a 6-well plate, followed by the addition of 30 mM d-glucose. Cells were then cultivated under aforementioned conditions for 48 h. After that, 0.25% trypsin digestion was performed, and cells were stained with Annexin V-FITC and propidium iodide (PI), followed by flow cytometry to separate apoptotic cells.

### Data analysis

Using data from at least 3 independent biological replicates, the mean values were calculated. Kruskal Wallis tests and post hoc Wilcoxon–Mann–Whitney tests were used to compare the differences among multiple groups. Comparisons between 2 groups were performed by unpaired *t* test. Correlations were analyzed by linear regression. *p* < 0.05 was considered as statistically significant.

## Results

### VIM-AS1 was downregulated in DR

**Fig. 1:**
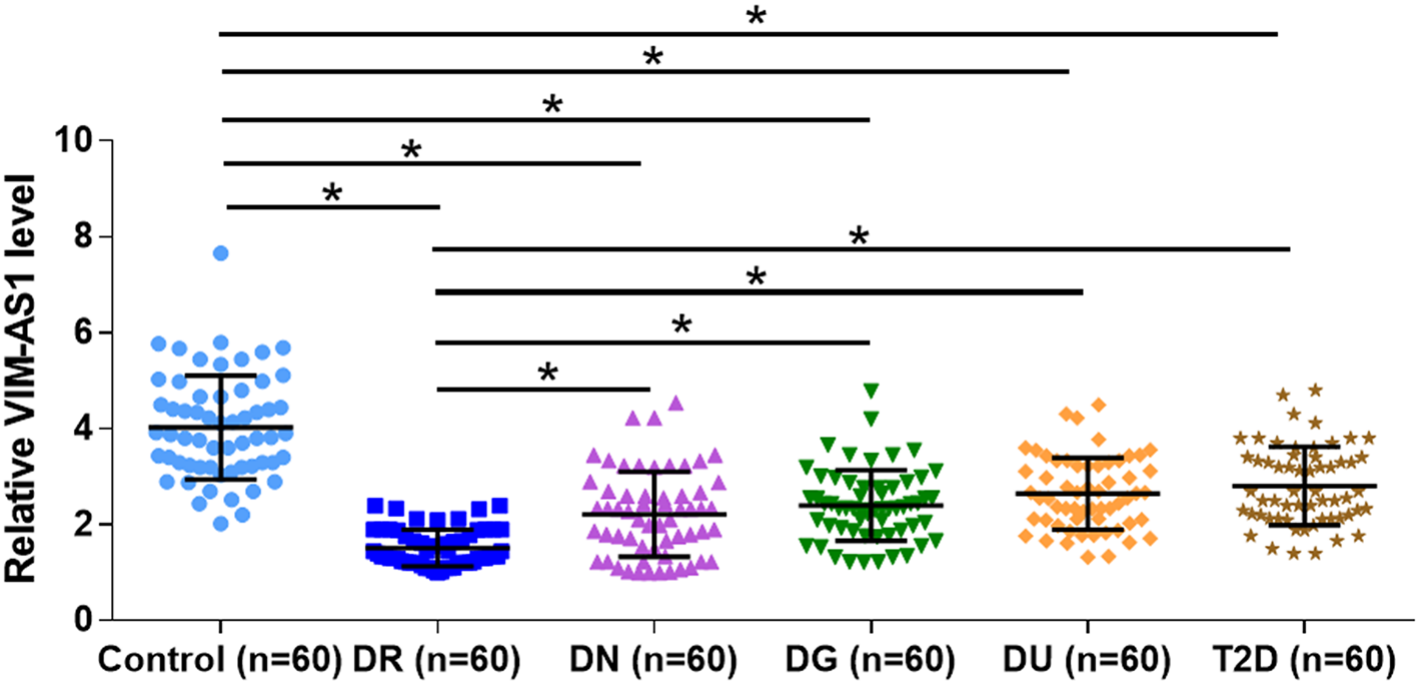
VIM-AS1 was downregulated in DR. Expression levels of in plasma samples from DR group (*n* = 60), DN group (*n* = 60), DG group (*n* = 60), DU group (*n* = 60), T2D group (*n* = 60) and control (*n* = 60) group were measured by qPCR. *p < 0.05

To evaluate the differential expressions of VIM-AS1 in diabetic complications, the expression levels of VIM-AS1 in plasma samples from DR group (*n* = 60), DN group (*n* = 60), DG group (*n* = 60), DU group (*n* = 60), T2D group (*n* = 60) and the control (*n* = 60) group were measured by qPCR. It was observed that the expression levels of VIM-AS1 were significantly lower in all diabetic groups in comparison with that in the control group (Fig. 1, *p* < 0.05). In addition, the expression levels of VIM-AS1 were the lowest in DR group in comparison with that in other diabetic groups (*p* < 0.05), indicating the possible involvement of VIM-AS1 in DR.

### VIM-AS1 can interact with miR-29 but they were not significantly correlated in DR patients

**Fig. 2:**
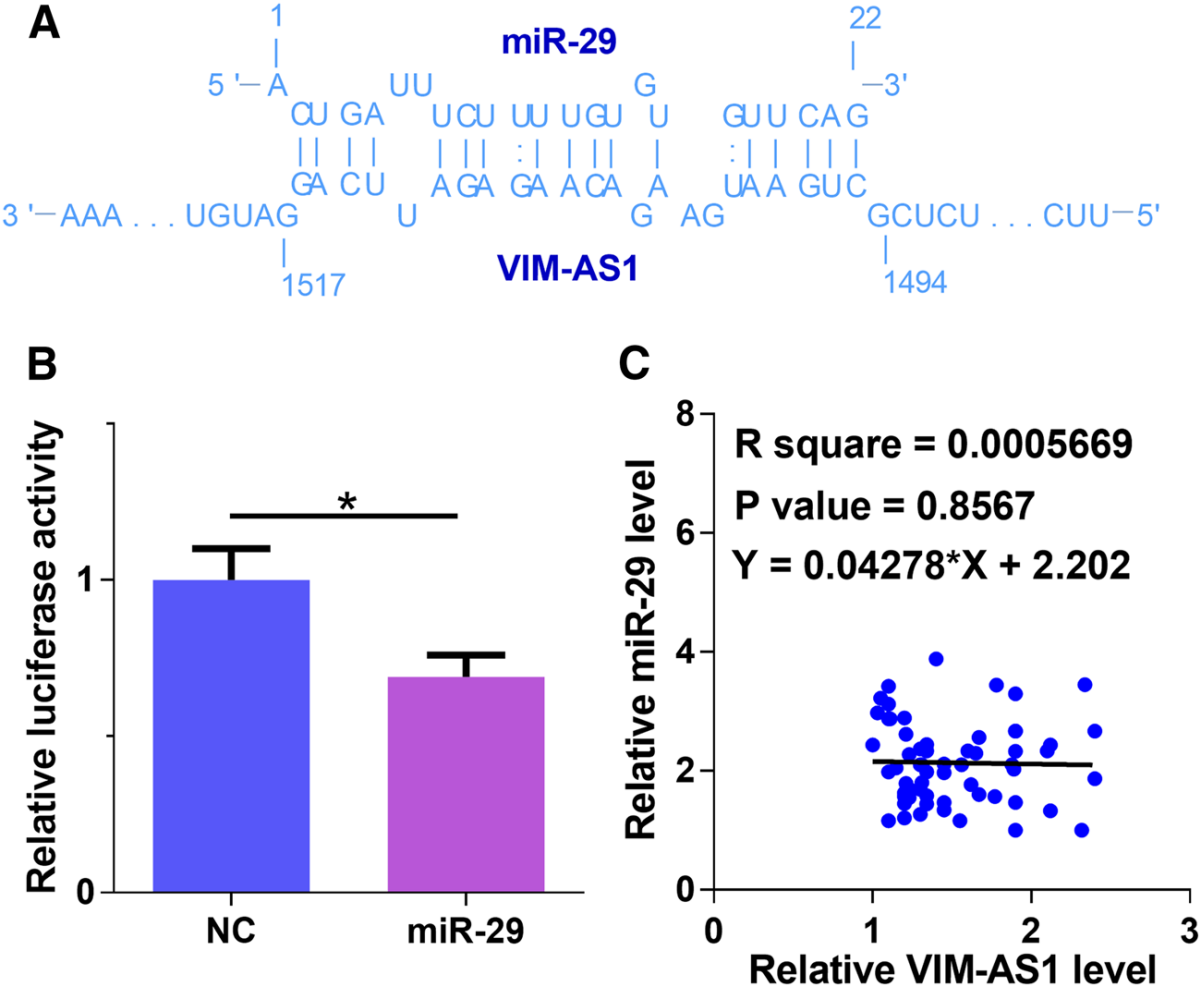
VIM-AS1 can interact with miR-29 but they were not significantly correlated in DR patients. The interaction between VIM-AS1 and miR-29 was analyzed by IntaRNA (https://rna.informatik.uni-freiburg.de/IntaRNA/Input.jsp). It was observed that miR-29 can form strong base pairing with VIM-AS1 (**a**). Dual luciferase assay was performed to further confirm the interaction between VIM-AS1 and miR-29 (**b**). Levels of miR-29 in plasma from DR patients were measured by qPCR, and the correlation between miR-29 and VIM-AS1 was analyzed by linear regression (**c**). *p < 0.05

The interaction between VIM-AS1 and miR-29 was analyzed by IntaRNA 2.0 (https://rna.informatik.uni-freiburg.de/IntaRNA/Input.jsp). It was observed that miR-29 can form strong base pairing with VIM-AS1 (Fig. 2a). Dual luciferase assay further confirmed the interaction between VIM-AS1 and miR-29. As shown in Fig. 2b, in comparison with NC group, the relative luciferase activity of miR-29 group was significantly lower (*p* < 0.05), indicating the regulating effect of VIM-AS1 on miR-29. The expression levels of miR-29 in plasma from DR patients were measured by qPCR, and the correlation between miR-29 and VIM-AS1 was analyzed by linear regression. It was observed that the expression of miR-29 and VIM-AS1 were not significantly correlated (Fig. 2c). Therefore, miR-29 and VIM-AS1 may not be able to regulate the expression of each other.

### VIM-AS1 and miR-29 did not affect the expression of each other

**Fig. 3:**
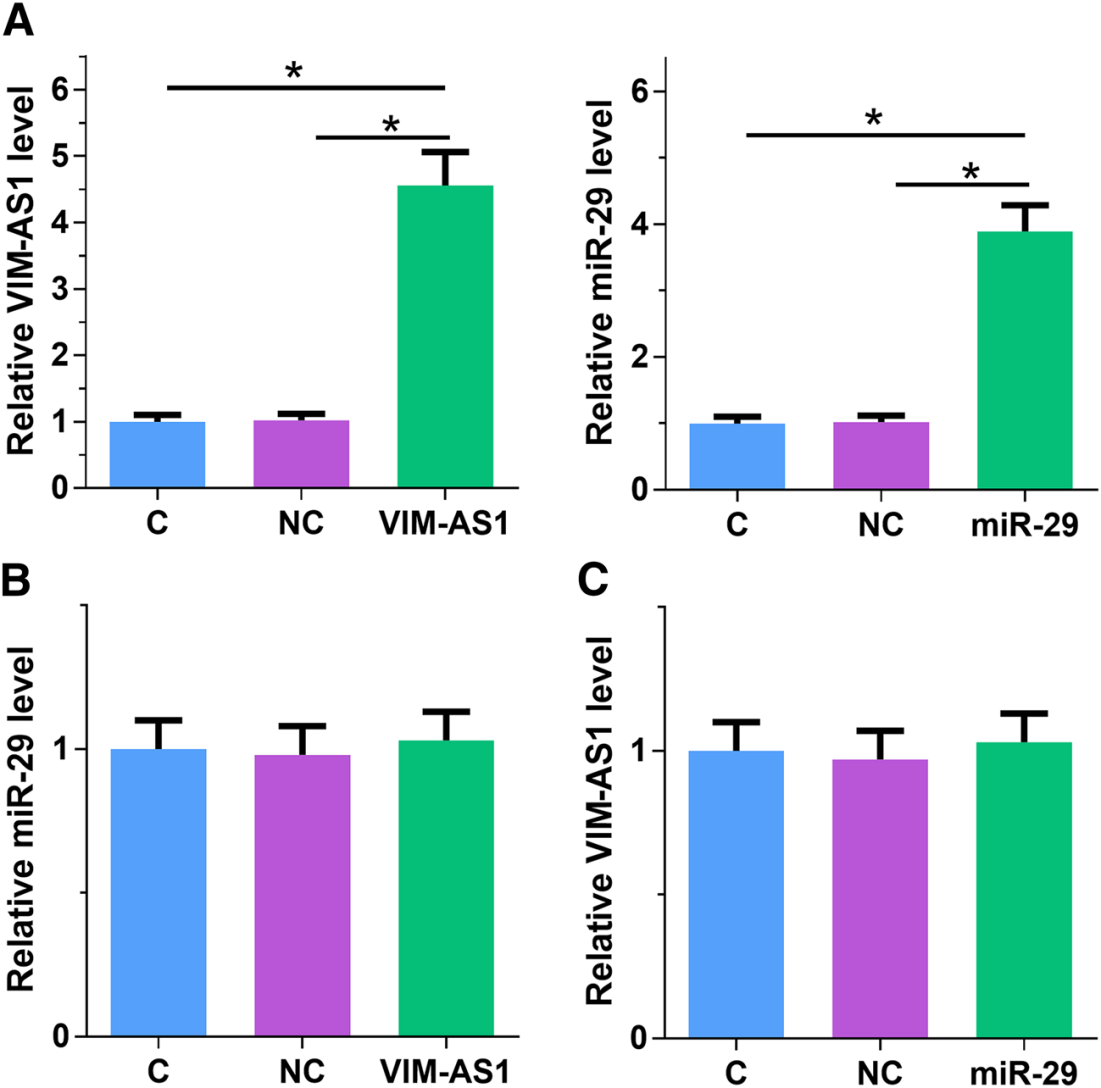
VIM-AS1 and miR-29 did not affect the expression of each other. H1RPE7 cells were transfected with VIM-AS1 expression vector and miR-29 mimic to further analyze the interactions between them. Overexpression of VIM-AS1 and miR-29 was confirmed by qPCR as 24 h post-transfection (**a**). The effects of overexpression of VIM-AS1 on miR-29 (**b**) and the effects of overexpression of miR-29 on VIM-AS1 (**c**) were analyzed by qPCR. Experiments were repeated 3 times and mean values were presented. *p < 0.05

To further explore the interaction between miR-29 and VIM-AS1, H1RPE7 cells were transfected with VIM-AS1 expression vector and miR-29 mimic to further analyze the interactions between them. Overexpression of VIM-AS1 and miR-29 was confirmed by qPCR as 24 h post-transfection (Fig. 3a, *p* < 0.05). In comparison with C and NC groups, overexpression of VIM-AS1 did not affect the expression of miR-29 (Fig. 3b). In addition, overexpression of miR-29 also did not affect the expression of VIM-AS1 (Fig. 3c). Therefore, VIM-AS1 is unlikely a target of miR-29.

### Overexpression of VIM-AS1 attenuated the effects of overexpressing miR-29 on glucose-induced h1RPE7 cell apoptosis

**Fig. 4:**
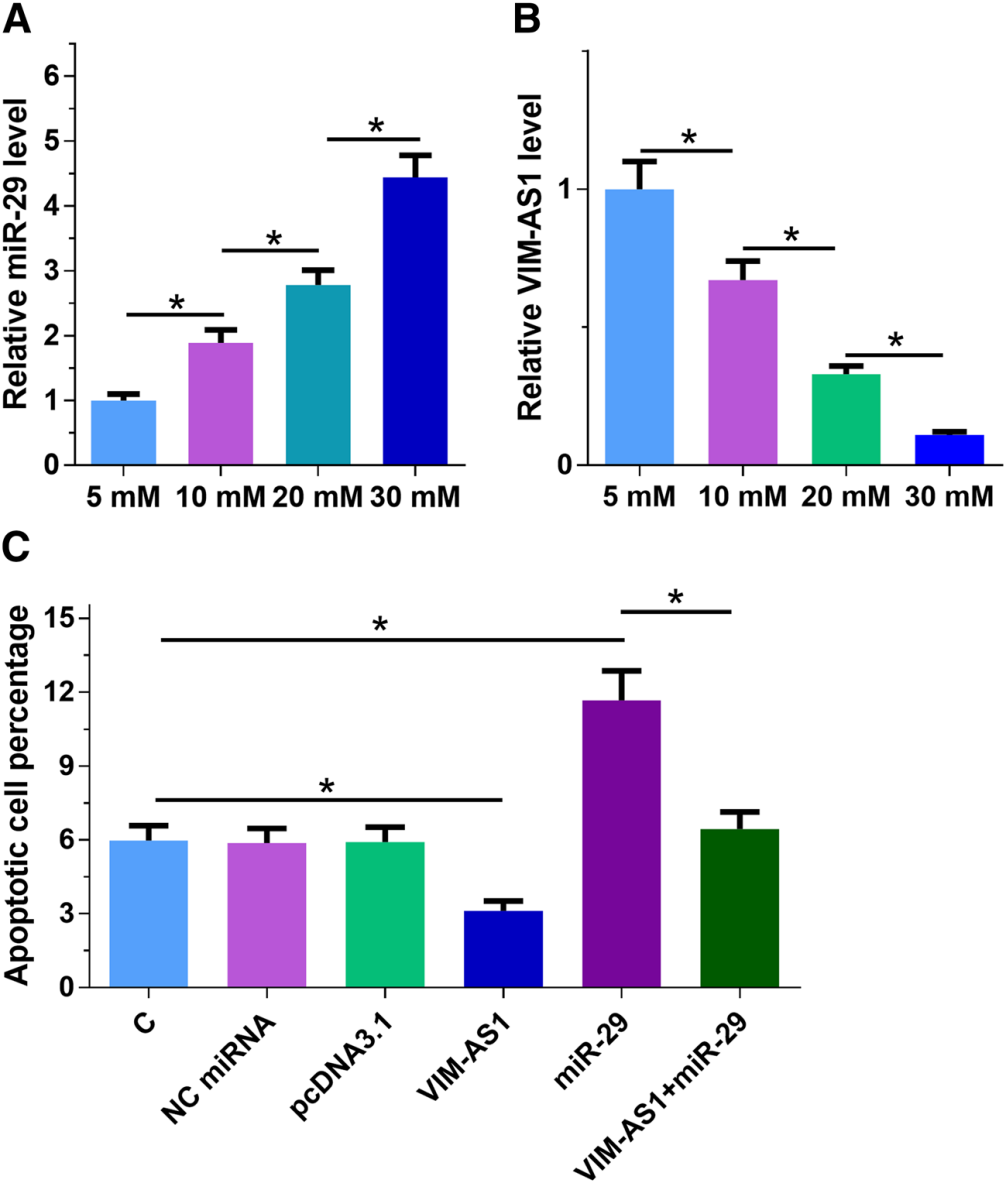
Overexpression of VIM-AS1 attenuated the effects of miR-29 on glucose-induced h1RPE7 cell apoptosis. H1RPE7 cells were cultivated in medium containing 5, 10, 20 and 30 mM d-glucose for 24 h, followed by the measurement of the levels of VIM-AS1 (**a**) and miR-29 (**b**) expression. Cell apoptosis analysis was performed to analyze the effects of overexpressing VIM-AS1 and miR-29 on h1RPE7 cell apoptosis during the treatment of 30 mM d-glucose (**c**). Experiments were repeated 3 times and mean values were presented. *p < 0.05

To analyze the effects of high glucose on the expression of VIM-AS1 and miR-29, H1RPE7 cells were cultivated in medium containing 5, 10, 20 and 30 mM d-glucose for 24 h, followed by measurement of the expression levels of VIM-AS1 and miR-29. It was observed that high glucose treatment led to upregulated miR-29 (Fig. 4a) and downregulated VIM-AS1 (Fig. 4b) in a dose-dependent manner. Cell apoptosis analysis was performed to analyze the effects of overexpressing VIM-AS1 and miR-29 on h1RPE7 cell apoptosis during the treatment of 30 mM d-glucose. In comparison with C and NC (NC miRNA or empty vector transfection) groups, overexpression of miR-29 led to increased cell apoptotic rate, while overexpression of VIM-AS1 played an opposite role and reduced the enhancing effects of overexpression of miR-29 on cell apoptosis (Fig. 4c, *p* < 0.05). Therefore, VIM-AS1 may suppression high glucose-induced apoptosis of h1RPE7 cells through miR-29.

## Discussion

The present study mainly investigated the functionality of VIM-AS1 in DR. We found that VIM-AS1 was downregulated in DR. In addition, this lncRNA might serve as an endogenous sponge of miR-29 and could attenuate its functions on glucose-induced h1RPE7 cell apoptosis.

A recent study reported a considerable number of differentially expressed lncRNAs in the pathogenesis of DR [13]. However, most of these lncRNAs have no critical roles in disease development and progression [13]. However, some studies indeed characterized lncRNAs as critical players in DR. For instance, lncRNA MALAT1 is overexpressed in DR, and it can regulate the miR-125b/VE-cadherin axis to participate in disease progression through neovascularization [14]. In another study, lncRNA HOTTIP was reported to be overexpressed in DR and can improve disease conditions by regulate p38-MAPK signaling [15]. A recent study reported the downregulation of VIM-AS1 in diabetic patients, while its roles in the development of diabetic complications are unclear. Our study confirmed the downregulation of VIM-AS1 in diabetic. We observed that the expression levels of VIM-AS1 were the lowest in DR patients in comparison with other diabetic groups, indicating its important roles in this type of diabetic complication.

Our study observed the downregulation of VIM-AS1 after high-glucose treatment. Therefore, the downregulation of VIM-AS1 in T2D patients is highly likely induced by the high glucose environment. However, the mechanism of the further downregulation of DR patients is unclear. It is possible that the formation of lesion in eyes may lead to the further downregulation of VIM-AS1 in plasma.

Interestingly, miR-29 can bind to VIM-AS1 but did not affect the expression of this lncRNA. Based on the findings in previous studies that lncRNAs could mimic miRNA targets to attenuate the functions of miRNAs [16, 17], we proposed that VIM-AS1 might be an endogenous sponge of miR-29. This speculation is supported by the observation that overexpression of VIM-AS1 reduced the effects of miR-29 on cell apoptosis.

In conclusion, VIM-AS1 is downregulated in DR and may sponge miR29 to inhibit high glucose-induced cell apoptosis. However, our mechanism may exist. Further investigations are still needed.
